# The psychometric property of a short-form of the Social Axioms Survey (SAS II)

**DOI:** 10.1186/s40359-023-01401-1

**Published:** 2023-11-07

**Authors:** Kwok Kit Tong, Juliet Honglei Chen, Mu He

**Affiliations:** 1 Department of Psychology, Faculty of Social Sciences, University of Macau, Macao, SAR China; 2 Department of Psychology, Zhejiang Sci-Tech University, Hangzhou, China

**Keywords:** Social axioms, Social belief, Short form, Personality, Cognitive flexibility, Interpersonal trust, Locus of control, Paranormal belief

## Abstract

**Background:**

Social Axioms are generalized beliefs and broad assumptions about the world, guiding behaviors across various social situations. Social Axioms are usually assessed by Social Axioms Survey II (SAS II). Nevertheless, the length of the scale may limit its usefulness in studies with strict time constraint. The present study aimed at developing a shorter version.

**Methods:**

A survey was conducted among 455 college students. First, we performed psychometric evaluation on the full item version of SAS II to identify items with superior psychometric properties for a brief version of SAS II. Second, we validated the psychometric properties of the brief version of SAS II.

**Results:**

A 20-item version of SAS II (SAS II-20) was developed, and it demonstrated adequate reliability and validity. The correlations between SAS II-20 and personality variables, cognitive flexibility, interpersonal trust, locus of control, and paranormal beliefs were consistent with past studies.

**Conclusions:**

SAS II-20 is psychometrically acceptable and provides a time-efficient measurement tool for investigating social beliefs.

## Background

In search of a framework to account for cultural variation in social behaviors, Leung et al. [1] advocated using generalized beliefs to complement value-based approaches. They argued that the generalized beliefs framework provided information that the value approaches could not detect, and it also provided triangulation for findings based on the value approaches [2, 3]. Leung and Bond [4] labeled such generalized beliefs (cf., beliefs that are specific to a set of actors, targets, and contexts) that are abstract enough to be applied across different contexts as Social Axioms, including individuals’ beliefs about themselves, the social and physical environment, and the spiritual world. Social Axioms are presented in the form of an assertion about the relationship between two entities or concepts [1]. Social Axioms are axiomatic because they are rarely questioned or elaborated upon [5] and moderately stable over time [6]. They are individuals’ broad assumptions and expectancies about contingencies in the world [7].

Based on the data from over 40 cultures, the five-factor structure of Social Axioms was proposed with good cross-cultural generalizability [4]. The five factors are *social cynicism* (a belief concerning negative human nature and social construct), *reward for application* (a belief concerning that the application of resources and effort will lead to positive outcomes), *social complexity* (a belief concerning flexible means to handle problems and to achieve outcomes), *fate control* (a belief concerning that fate is predetermined by external factors), and *religiosity* (a belief in the spiritual forces and religious institutions; [1, 8]). Social Axioms have been applied to explain a range of social behaviors in the domains of social relations [9–11], learning and education [12, 13], organizational psychology [2, 14, 15], and health and well-being [16–21].

Using an inductive approach, Leung et al. [1] originally created the measurement of Social Axioms based on literature review, content analysis (e.g., newspapers, magazines, popular songs, textbooks, poetry, and proverbs), as well as open-end questionnaires and interviews about people’s most important principles and major beliefs that guide their interactions with others and everyday matters (e.g., self attributes, psychological attributes, social attributes, group attributes, nature, and supernature) in various life domains (e.g., health, love, family, politics, and religion). More than 2000 statements were identified, and 182 items were retained. Based on the data from Germany, Hong Kong, Japan, and Venezuela, five common factors were identified. To make the measurement concise for application, items with low factor loadings were removed, and finally 60 items were retained. The 60-item Social Axioms Survey (SAS) has been validated across 41 cultural groups [8].

A deductive approach was adopted to create Social Axioms Survey II (SAS II) to address the low reliability of fate control and social complexity dimensions [6]. Employing a culturally decentered approach, they invited psychologists from 10 countries with different religions, political systems, and socioeconomic development to generate items based on SAS dimensions. Leung et al. [6] pooled 143 new items with 39 pan-cultural items from SAS and tested the items in 11 countries. The long version of SAS II contains 83 items, and the 40-item short version of SAS II was created based on item loadings. SAS II was shown to be stable over time at the societal level [6] and the individual level [20, 22, 23].

Social Axioms are applicable to various issues and behaviors, and a shorter scale may help enhance the efficacy in research [6, 8]. Referring to psychometric analysis in a variety of domains, a shorter scale does not necessarily inferior to the full version [24–27]. While long questionnaires may trigger potential biases [28], a brief one may be helpful when there is a strict time limitation. The issue of questionnaire length looms large when a great number of participants have to be contacted. For example, in large-scale surveys that need to address a number of environmental and health issues (e.g., surveys conducted during the COVID-19 pandemic), only a tiny portion of the survey time can be allocated to assess individual characteristics. In addition, a long survey may also lead to fatigue and careless responses [29].

In order to increase survey efficiency and facilitate Social Axioms related research, the present study aimed to construct a briefer version of the Social Axioms Survey based on the 40-item SAS II. We expected item response theory-based analysis can provide additional information for identifying items that contribute most to discriminating different levels of the latent variable of interest [30, 31]. Once the short form is composed, we hypothesized that it could display a similar five-factor structure as the 40-item SAS II and measurement invariance across genders. In addition, we hypothesized that our short-form of SAS could display psychometric soundness in terms of reliability and criterion validity, similar to previous studies on SAS II [6]. In the current study, we specifically included Interpersonal Trust, Cognitive Flexibility, Locus of Control, Paranormal Beliefs, and Big Five personality traits as indicators of criterion validity because Social Axioms have consistently displayed significant associations with these constructs cross-culturally [6, 32].

## Method

### Procedure and participants

We conducted an online survey among Chinese college students in a public university in China. Informed consent was obtained from each participant before they took the survey. Four hundred and fifty-five participants completed the survey voluntarily without monetary incentive. We added four attention check items in the survey to screen out the participants who responded carelessly (e.g., “This item is for attention check, please select strongly agree”). The data of 20 participants were discarded for failing the attention check, retaining a sample size of 435 for formal analyses. Among this sample, 39.8% were male (95% *CI* [35.2%, 44.4%]), and 60.2% were female (95% *CI* [55.6%, 64.8%]). Their age ranged from 18 to 25 years (*M* = 19.88, *SD* = 1.51). The ethical approval for the present study was obtained from the Ethics Committee of Department of Psychology, University of Macau (Approval Code: 2022-04).

### Measures

#### Social axioms

The 40-item SAS II [6] was used as the starting point for developing a short-form measure of Social Axioms in the present study. It includes five 8-item factors to capture individuals’ generalized beliefs regarding social cynicism, reward for application, social complexity, fate of control, and religiosity (see Table 1). All items are rated on a 5-point scale (1 = *strongly disagree* to 5 = *strongly agree*). A higher total score indicates stronger belief. The 40-item SAS II displayed satisfactory reliability for each Social Axioms factor in the present sample: 0.79 (social cynicism), 0.82 (reward for application), 0.86 (social complexity), 0.82 (fate control), and 0.83 (religiosity).

**Table 1:** Item-total correlation of the 40 items in SAS II (*N* = 435)

Item	Item-total correlation
Social Cynicism	
1. People create hurdles to prevent others from succeeding.	0.50
2. People dislike others who succeed in life.	0.46
3. Powerful people tend to exploit others.	0.49
4. People who become rich and successful forget the people who helped them along the way.	0.45
5. Kind-hearted people usually suffer losses.	0.58
6. Opportunities for people to get wealthy promote dishonesty.	0.57
7. Kind-hearted people are easily bullied.	0.55
8. The only way to get ahead is to take advantage of others. **	0.37
Reward for Application	
1. One will succeed if he/she really tries.	0.49
2. Success requires strong willpower.	0.57
3. Hard-working people are well rewarded.	0.55
4. Adversity can be overcome by effort.	0.64
5. Difficult problems can be overcome by hard work and persistence.	0.68
6. Hard working people will achieve more in the end.	0.59
7. Endurance and determination are key to achieving goals.	0.68
8. Building the way step by step leads to success. **	0.22
Social Complexity	
1. There is usually more than one good way to handle a situation.	0.58
2. A person’s behavior is influenced by many factors.	0.71
3. People can suddenly lose everything they have.	0.66
4. Many issues appear far more complicated than they really are.	0.71
5. People with different opinions can all be correct.	0.56
6. People may have opposite behaviors on different occasions.	0.64
7. One has to deal with matters according to the specific circumstances.	0.64
8. A bad situation can suddenly change for the better. **	0.39
Fate Control	
1. Fate determines one’s successes and failures.	0.55
2. Fate determines a person’s success in life.	0.61
3. Matters of life and death are determined by fate.	0.61
4. There are ways for people to find out about their fate.	0.56
5. The people whom a person will love in his or her life is determined by fate.	0.51
6. Individual characteristics, such as appearance and birthday, can reveal one’s fate.	0.58
7. Luck can be enhanced by certain tactics.	0.49
8. There are certain ways for people to improve their destiny. **	0.39
Religiosity	
1. Belief in a religion helps one understand the meaning of life.	0.65
2. Religious faith contributes to good mental health.	0.69
3. Belief in a religion makes people good citizens.	0.57
4. Religion makes people healthier.	0.69
5. Religion helps people make good choices for their lives.	0.72
6. Religion makes people happier.	0.67
7. Religion slows down human progress. **	0.21
8. There is a supreme being controlling the universe. **	0.32

*Note.* Items labeled with ** were removed

#### Interpersonal trust

The 25-item Interpersonal Trust Scale [33] was used to measure interpersonal trust. Example items are “Parents usually can be relied upon to keep their promises” and “In dealing with strangers, one is better off to be cautious until they have provided evidence that they are trustworthy”. Each item is rated on a 5-point scale (1 = *strongly disagree* to 5 = *strongly agree*). A higher total score indicates a higher level of interpersonal trust. The reliability in the present study was adequate with Cronbach’s *α* = 0.75.

#### Cognitive flexibility

The 13-item Cognitive Flexibility Scale [34] was used to assess cognitive flexibility. A sample item is “I can communicate an idea in many different ways”. Items are rated on a 6-point scale (1 = *strongly disagree* to 6 = *strongly agree*). A higher total score indicates greater cognitive flexibility. The Cronbach’s *α* was 0.84 in the present study.

#### Locus of control

Locus of control was assessed by the 23-item Locus of Control Scale [35]. This scale consists of 23 forced-choice pairs, with one internally oriented statement (rated as “0”) and the other externally oriented statement (rated as “1”). A sample item is “People’s misfortunes result from the mistakes they make” vs. “Many of the unhappy things in people’s lives are partly due to bad luck”. A higher total score indicates a higher level of external locus of control. The reliability (KR-20) in the present study was 0.69.

#### Paranormal beliefs

In the present study, we adopted paranormal beliefs as indicators to evaluate the criterion validity of the Religiosity and Fate Control axiom. According to the domain and content described in the Religiosity and Fate Control items, four subscales (i.e., Traditional Religious Belief, Superstition, Spiritualism, and Precognition) of the Revised Paranormal Belief Scale [36] were used for measuring paranormal beliefs in the present study. Other paranormal belief subscales (i.e., Psi, Witchcraft, and Extraordinary Life Forms) were not included in the present study as they were less related to the construct measured by Religiosity and Fate Control. In addition, these subscales might not represent the typical paranormal beliefs among Chinese (e.g., black magic, witches), and they require substantial adaptation before they can be used. The items were rated on a 7-point scale from 1 = *strongly disagree* to 7 = *strongly agree*. A sample item is “The soul continues to exist though the body may die”. A higher total score indicates stronger paranormal beliefs. In this study, the Cronbach’s *α* was 0.95.

#### Big five personality

The 20-item Mini-International Personality Item Pool [25] was used for evaluating Big Five Personality. There are four items for each personality factor. Sample items are: “I have frequent mood swings (Neuroticism)”, “I am the life of the party (Extraversion)”, “I have a vivid imagination (Intellect)”, “I sympathize with others’ feelings (Agreeableness)”, and “I get chores done right away (Conscientiousness)”. Each item is rated on a 5-point scale from 1 = *strongly disagree* to 5 = *strongly agree*. In the present study, the Cronbach’s α of each personality factor was 0.73 (Neuroticism), 0.76 (Extraversion), 0.65 (Intellect), 0.70 (Agreeableness), and 0.60 (Conscientiousness).

### Statistical analyses

#### Identify items for a brief version of SAS II

We conducted a psychometric evaluation on all 40 items in SAS II to identify items with superior psychometric properties. Items were first screened with corrected item-total correlation. According to the suggestion of Hair et al. [37], we eliminated items with an item-total correlation less than 0.40. Then we conducted item response theory (IRT)-based analysis. The two-parameter logistic model was used to fit the items in each social axiom dimension. The discrimination parameter (*a*) and the location parameter (*b*) were evaluated, and the inlier-pattern-sensitive fit statistic (Infit) and outlier-sensitive fit statistic (Outfit) were used as the main indicators of the fitness of each item. According to the suggestion of Bond et al. [38], Infit and Outfit values within 0.6–1.4 are considered acceptable. In addition, root mean square error of approximation of *S-X*^*2*^ statistic (RMSEA.*S-X*^*2*^) was adopted as a supplementary statistic to determine item fitness with the advantage of eliminating the interference of sample size, and lower RMSEA.*S-X*^*2*^ values indicate better fitness [39]. IRT-based analysis was conducted using the Mirt Package in R [40].

#### Validate the psychometric properties of the brief SAS II

First, the measurement reliability of the brief SAS II was tested with composite reliability higher than 0.60 as adequate [41]. Second, the accuracy of the brief SAS II was evaluated by calculating the intraclass correlation coefficient with the 95% confidence interval for agreement between the brief version and full item version [42]. Third, confirmatory factor analysis (CFA) was conducted to test whether the brief SAS II can fit the original five-factor structure of SAS. The CFA model would be accepted if it meets the following criteria: (1) relative Chi-square value (*χ*^*2*^*/df*) < 3.0; (2) Comparative Fit Index (CFI) ≥ 0.90; (3) Tucker-Lewis Index (TLI) ≥ 0.90; (4) Goodness-of-fit Index (GFI) ≥ 0.90; (5) Parsimony Comparative Fit Index (PCFI) ≥ 0.50; (6) Parsimony Goodness-of-fit Index (PGFI) ≥ 0.50; (7) Parsimony Normed Fit Index (PNFI) ≥ 0.50; (8) Root Mean Square Error of Approximation (RMSEA) ≤ 0.08; and (9) Standardized Root Mean Square Residual (SRMR) ≤ 0.08 [43–45]. A scale item is accepted when its factor loading is higher than 0.40 [41]. Fourth, measurement invariance between genders was tested under the framework of multiple-group CFA [46] and based on a nonsignificant *Δχ*^*2*^ (*p* < .05; [47]) at the configural (identical factor structures), metric (equality of factor loadings), and scalar (equality of item intercepts) level. Last, bivariate correlation analyses were conducted to evaluate the criterion validity of the brief SAS II by testing its association with variables (i.e., interpersonal trust, cognitive flexibility, paranormal beliefs, locus of control, and big five personality factors) that have been previously shown to be related to social axiom dimensions.

## Results

### Identify items for a brief version of SAS II

#### Item-total correlation

The item-total correlation coefficients of the 40 items in SAS II were shown in Table 1. Six items with an item-total correlation less than 0.40 (labeled with **) were eliminated from following analyses.

#### Item response theory-based analysis

The result of IRT-based analysis was displayed in Table 2. Most items had desired Outfit and Infit values, except for “Reward for Application 2”. We then evaluated the model fitness of each item according to the RMSEA.*S-X*^*2*^ value. In each social axiom dimension, four items with the smallest RMSEA.*S-X*^*2*^ values were selected to compose a 20-item version of the SAS II (SAS II-20).

**Table 2:** IRT fit statistics of the SAS items (*N* = 435)

Item	Outfit	Infit	RMSEA.*S-X*^*2*^
Social Cynicism 2	0.99	0.98	.000
Social Cynicism 6	0.87	0.87	.001
Social Cynicism 4	0.97	0.97	.018
Social Cynicism 7	1.14	0.86	.029
Social Cynicism 3**	0.97	0.95	.030
Social Cynicism 5**	0.83	0.82	.030
Social Cynicism 1**	0.91	0.91	.052
Reward for Application 5	0.91	0.80	.014
Reward for Application 4	0.93	0.96	.020
Reward for Application 7	0.71	0.75	.023
Reward for Application 6	0.88	0.92	.027
Reward for Application 1**	1.12	1.03	.033
Reward for Application 3**	0.93	0.93	.039
Reward for Application 2**	2.60	1.00	.065
Social Complexity 2	0.69	0.82	.038
Social Complexity 6	0.87	0.96	.043
Social Complexity 7	1.02	1.02	.046
Social Complexity 4	0.96	0.84	.059
Social Complexity 3**	1.02	0.97	.063
Social Complexity 1**	1.06	0.96	.064
Social Complexity 5**	1.07	1.02	.073
Fate Control 3	0.97	0.78	.011
Fate Control 2	0.81	0.83	.025
Fate Control 6	1.01	0.96	.030
Fate Control 1	0.99	0.92	.030
Fate Control 5**	0.99	0.97	.031
Fate Control 7**	1.04	1.00	.032
Fate Control 4**	1.01	0.98	.042
Religiosity 5	0.85	0.85	.000
Religiosity 4	0.82	0.83	.021
Religiosity 3	0.95	0.98	.022
Religiosity 1	0.91	0.89	.039
Religiosity 6**	0.81	0.92	.041
Religiosity 2**	0.89	0.87	.045

*Note.* Infit = inlier-pattern-sensitive fit statistic. Outfit = outlier-sensitive fit statistic. RMSEA.*S-X*^*2*^ = Root mean square error of approximation of *S-X*^*2*^ statistic. Items labeled with ** were removed

### Validate the psychometric properties of the SAS II-20

#### Reliability and accuracy analysis

The composite reliabilities of SAS II-20 factors were adequate with social cynicism 0.66, reward for application 0.83, social complexity 0.82, fate control 0.78, and religiosity 0.82. Moreover, we compared the internal consistency reliability (Cronbach’s α) of SAS II-20 with SAS II-40. Except for social cynicism (0.65 vs. 0.79), all of the other factors did not show apparent decrease in internal consistency reliability (reward for application 0.82 vs. 0.82, social complexity 0.82 vs. 0.86, fate control 0.77 vs. 0.82, and religiosity 0.81 vs. 0.83). The intraclass correlations of SAS II-20 factors with the full item version factors were high, ranging between 0.93 (95% *CI* [0.92, 0.93]) and 0.96 (95% *CI* [0.95, 0.97]).

#### Confirmatory factor analysis

The result of CFA showed that, except for TLI = 0.88, most model fit indices were adequate, with *χ*^*2*^ (160) = 461.89, *p* < .001, *χ*^*2*^*/df* = 2.89, CFI = 0.90, GFI = 0.90, PCFI = 0.76, PGFI = 0.69, PNFI = 0.72, SRMR = 0.06, and RMSEA = 0.07, implying that SAS II-20 could fit the five-factor structure of SAS. The standardized factor loadings of all SAS II-20 items were satisfactory, ranging from 0.42 to 0.82.

#### Gender invariance analysis

The result of multiple-group CFA between genders was shown in Table 3. The model fit indices of configural and metric invariance models were acceptable, and the significance tests of model comparison further suggested that the models met the invariance criterion, indicating SAS II-20 can be used invariably across genders with similar factor structures and factor loadings. The scalar invariance model firstly showed inadequate fit indices, and the model comparison test showed a significant result, implying the model did not meet the invariance criterion. We then tested a partial scalar invariance model by relaxing the constraints on four item intercepts with significant critical ratios (social cynicism 4, reward for application 6, social complexity 4, and religiosity 4). The partial scalar invariance model showed satisfactory fit indices, and the significance test of model comparison indicated the model met the invariance criterion.

**Table 3:** Gender invariance analysis on SAS II-20 (*N* = 435)

Model	Model fit					Model comparison		
	*χ* ^*2*^ */df*	CFI	RMSEA	SRMR		*Δχ* ^*2*^	*Δdf*	*p*
Configural invariance	2.01	0.90	0.05	0.08				
Metric invariance	1.98	0.90	0.05	0.08		18.21	15	0.252
Scalar invariance	1.99	0.89	0.05	0.09		42.24	20	0.003
Partial scalar invariance	1.95	0.90	0.05	0.08		22.78	16	0.120

*Note*: CFI = Comparative Fit Index, RMSEA = Root Mean Square Error of Approximation, SRMR = Standardized Root Mean Square Residual

#### Criterion validity analysis

In line with our expectation, a series of significant correlations were observed between the social axiom dimensions of SAS II-20 and relevant variables (see Table 4). Social cynicism was positively correlated with locus of control, and negatively correlated with interpersonal trust and agreeableness. Reward for application was positively correlated with agreeableness and conscientiousness, and negatively correlated with locus of control. Social complexity was positively correlated with cognitive flexibility and intellect. Fate control was positively correlated with locus of control and paranormal beliefs. Religiosity was positively correlated with paranormal beliefs and interpersonal trust. The five dimensions of SAS II-20 demonstrated adequate criterion validity.

**Table 4:** Bivariate correlation analyses (*N* = 435)

Variables	Social Cynicism	Reward for Application	Social Complexity	Fate Control	Religiosity
Interpersonal Trust	− 0.46^***^	0.12^*^	− 0.28^***^	− 0.14^**^	0.15^**^
Cognitive Flexibility	− 0.10	0.37^***^	0.32^***^	− 0.05	0.10^*^
Locus of Control	0.31^***^	− 0.28^***^	0.06	0.21^***^	− 0.08
Paranormal Beliefs	0.18^***^	0.02	− 0.09	0.52^***^	0.26^***^
Neuroticism	0.12^*^	− 0.05	0.07	0.13^**^	− 0.02
Extraversion	− 0.11^*^	0.13^**^	− 0.07	0.11^*^	0.01
Intellect	− 0.11^*^	0.09	0.22^***^	− 0.11^*^	0.04
Agreeableness	− 0.16^***^	0.36^***^	0.25^***^	0.05	0.10^*^
Conscientiousness	− 0.19^***^	0.27^***^	0.16^**^	− 0.18^***^	0.07

*Note*: ^*^p < .05; ^**^p < .01; ^***^p < .001

## Discussion

While SAS and SAS II have been shown to be helpful in predicting a range of behaviors [11, 13, 14, 17, 18, 48, 49], the length of the questionnaire may substantially limit its applicability. In studies that require to collect large community samples (e.g., public health issue), survey time and cost are usually major research constraints [50]. Researchers may have to give up measuring generalized social beliefs or select one or two social axiom dimensions for their studies. In studies utilizing online surveys and phone surveys that participants can break off or mid-terminate the study easily, a short version of questionnaire would help reduce fatigue and facilitate a higher completion rate. Based on our estimation, the 40-item version of SAS II takes about four minutes to complete, while the SAS II-20 takes about two minutes. The present study offers an additional choice to researchers so that they can choose between different versions of SAS II after considering the tradeoffs according to the particular conditions and scenarios of their research project.

The present study selected four items from each of the five social axiom dimensions based on the inlier-pattern-sensitive fit statistic, outlier-sensitive fit statistic, and root mean-square error of approximation of *S-X*^*2*^ statistic. The resulting 20-item scale showed an adequate fit to a five-factor structure. The reliability of SAS II-20 was comparable to the original SAS II [6]. SAS II-20 achieved configural and metric invariance between genders. However, partial scalar invariance can be established only after four items were released (one item from each SAS dimension except fate control). Given that the original study did not test measurement invariance across genders, we cannot conclude whether the findings were related to the items themselves or the characteristics of our sample. Therefore, we suggest that gender difference in social axiom dimensions should be interpreted cautiously.

The SAS II-20 demonstrated satisfactory psychometric properties, and we believe it can be a useful complement to the original version of SAS II. The associations of five social axiom dimensions in SAS II-20 with Big Five Personality factors were generally consistent with Leung et al. [6]. Our findings showed that people who believe in reward for application may be more conscientious and agreeable; social complexity was positively linked to intellect; social cynics had a slightly lower level of agreeableness. The present study provided additional information concerning the relationship between fate control and Big Five factors. Leung et al. [6] reported a possible relationship between fate control and neuroticism, and our study observed a mildly positive association. In addition, consistent with Singelis et al. [32], people holding stronger social complexity beliefs showed more cognitive flexibility and less interpersonal trust. Similarly, people who had a higher level of social cynicism beliefs tended to have weaker interpersonal trust and stronger external locus of control. In addition, those who believe in fate control may be more likely to have paranormal beliefs and external locus of control. Singelis et al. [32] predicted but did not find a relation between reward for application and internal locus of control while the relation was found in the present study. Moreover, in line with previous findings [51, 52], people holding stronger religiosity beliefs displayed a slightly higher level of interpersonal trust and paranormal beliefs in the present study. In short, the associations between the five dimensions of SAS II-20 and outcome variables were generally consistent with past studies.

The present study has its limitations. First, the reliability of social cynicism was not high, similar to the finding in the original study on SAS II [6]. It may result from the heterogeneity of the content (various beliefs in different social domains). Future research would be useful to establish the test-retest reliability to assess this dimension [53]. Second, similar to most social axiom studies, the present study relies on self-report data. SAS and SAS II have not been utilized in third party assessment (e.g., peer ratings), and hence response biases (e.g., social desirability bias) may affect the accuracy of the measurement. Third, the present study used a cross-sectional design, which could not test causal effects. Therefore, the findings on the relations between social axiom dimensions and criterion variables have to be interpreted with caution. Fourth, considering that paranormal beliefs may be culturally bounded, the current study did not include the full paranormal beliefs scale. The validity of the Religiosity and Fate Control social axiom dimensions in other cultures would need to be verified in additional research. In cross-cultural studies, we should be cautious in interpreting findings concerning these two social axiom dimensions.

## Conclusions

The present study selected 20 items from the 40-item version of SAS II, and the resulting scale has adequate psychometric properties to be used in research investigating Social Axioms. The SAS II-20 provides an additional option for researchers to optimize the design of surveys depending on different research needs. We believe that the new short form’s benefits would outweigh its costs in a variety of situations that avoid respondents’ fatigue and promote time-efficient data collection.

## Data Availability

The data that support the findings of the present study are available from the corresponding author on reasonable request. The authors declared no restrictions on research materials availability.
