# Publisher Correction: The psychometric property of a short-form of the Social Axioms Survey (SAS II)

**DOI:** 10.1186/s40359-023-01461-3

**Published:** 2023-12-06

**Authors:** Kwok Kit Tong, Juliet Honglei Chen, Mu He

**Affiliations:** 1 Department of Psychology, Faculty of Social Sciences, University of Macau, Macao, SAR China; 2 Department of Psychology, Zhejiang Sci-Tech University, Hangzhou, China

**BMC Psychology (2023) 11:377**

10.1186/s40359-023-01401-1

Following publication of the original article [1], it came to the journal’s attention that the name of the author Mu He had been duplicated in the author list. This technical error has since been fixed. The publisher thanks you for reading this erratum and apologizes for any inconvenience caused.

